# Poking Fun at the Matrons

**Published:** 1916-04-01

**Authors:** 


### THE MATRONS' AND SISTERS' DEPARTMENT.

### "POKING FUN AT THE MATRONS."

Since the war began the ever-increasing demand upon our time and energy to lend a hand, give counsel, inspect hospitals, encourage the workers, and in various other ways to do our part during the war, have given us a unique opportunity to know the facts and appreciate with heartfelt gratitude the magnificent output of personal service by those women of our country and Empire who have devoted their time and energies to provide for the wounded and the sick. The quality of this personal service, which has entailed in the case of many matrons and sisters a working day of continuous hours (with a tendency to increase constantly in number), cheerfully and efficiently occupied for the patients in hospitals of every degree, is one of the most inspiring and uplifting forces created by the war. Before the war a matron of a large hospital who was competent and conscientious in her work was often overdone by the strain. We know a number of matrons of this type who since the war have voluntarily undertaken the additional duties of principal matron of a large Territorial or similar hospital, and have risen to the occasion with remarkable courage and devotion. The quality of this service is demonstrated by the further fact, of which we have incontrovertible evidence and knowledge, that, taken as a whole, the results, as represented by the condition and efficiency of the hospitals of all types where our warriors have been received as patients, are wonderful. The few exceptions which have so far manifested themselves do but emphasise the splendid results in the case of the majority. The extra accommodation provided in this way must far exceed 100,000 beds. Being from circumstances familiar with these facts, we are confident that history will record that our matrons, sisters, and nurses, like our warriors, have given and are giving whole-heartedly of their best in the present hour of peril for nation, Empire, and people.

When the vision of this splendid outpouring of personal service by British women workers among the sick suddenly came to us with overwhelming force, we were filled with the desire successfully to create an album to contain the portraits of all these noble women who have served as matrons during the time of the great war. In The Hospital last week we dwelt on the historical value of such an album. This called forth the remark that matrons might consider the Editor was poking fun at them. Could the absence of imagination and apprehension such criticism exhibits be more remarkable? Was it due to war pressure, or overwork, or to that attractive modesty for which the best of women are so famous? Anyway, the portraits, we are glad to say, are coming in, and we hope every matron will co-operate to make this album comprehensive and complete.

